# Public Health Care Financing and the Costs of Cancer Care: A Cross-National Analysis

**DOI:** 10.3390/cancers10040117

**Published:** 2018-04-12

**Authors:** Ana Iolanda Voda, Ionel Bostan

**Affiliations:** 1 Department of Interdisciplinary Research—Humanities and Social Sciences, Alexandru Ioan Cuza University of Iasi, 700107 Iasi, Romania; yolanda.voda@gmail.com; 2 Faculty of Law and Administrative Sciences, “Stefan cel Mare” Univesity, 720229 Suceava, Romania

**Keywords:** public health, cancer expenditure, public financing

## Abstract

Expenditure and financing aspects in the healthcare system in general, and in cancer care in particular, are subjects of increasing concern to the medical community. Nowadays, it is imperative for the healthcare system to respond to the challenge of universal access to quality healthcare, by measuring the financial resources within the healthcare sector. The purpose of this review is to highlight the major gaps in the healthcare expenditures for all types of care, as well as on cancer and anti-cancer drugs across 28 European Union member states. The indicators taken into account are divided into two major groups: (1) healthcare expenditures for all types of care, and (2) healthcare expenditures on cancer and anti-cancer drugs. The programs used for our analysis are SPSS Statistics V20.0 (IBM Corporation, Armonk, NY, USA) and Stat World Explorer. The overall picture confirms that there are considerable disparities between the 28 countries in relation to their expenditures on health. The trend in public expenditures for all types of care, compared to the share of healthcare expenditures as a percentage of the GDP, shows the increase of health expenses between 2010 and 2014, but a lower rise compared to the total GDP increase. Healthcare expenditure on cancer (%THE) is rather low, despite the high cost associated with anti-cancer drugs. New treatments and drugs development will be increasingly difficult to achieve if the share devoted to cancer does not increase, and the lack of funds may act as a barrier in receiving high-quality care.

## 1. Introduction

In the recent years, due to innovative technologies, continuous personnel development of healthcare professionals, treatment patterns, and changing diagnostics, cancer-related expenditures have risen, and are expected to rise faster than any other related area of healthcare [1]. The American Society of Clinical Oncology acknowledged the rising economic burden of cancer patients compared with other chronic illnesses [2]. Across the European Union (EU), the World Health Organization Report (2016) showed a similar scenario: after cardiovascular diseases, cancer causes the second greatest burden on patients [3].

Many specialists (Table 1) in the field [1,2,3,4,5,6,7], using different approaches and variables, provide significant details and information about how changes in the burden of disease and innovative treatments, screening, and prevention are reflected in the cost of cancer. For instance, Bernard et al. (2011) [8] estimates the out-of pocket burden among non-elderly adults with cancer. Using a panel survey of people 18 to 64 years old, the authors provide important information about medical expenditure, insurance coverage, and other socioeconomic patient characteristics. Their main finding reveals that non-elderly adults with cancer are more likely to have high out-of-pocket burdens compared with other chronically ill patients. Gielen et al. (2010) [4] examine different patterns of healthcare use and expenditure in the last 6 months of life within cancer and non-cancer patients in Belgium. The main variables used in the analysis were number of transitions (place of stay and place of death), hospital days, home care services, number of days with at least one contact with a general practitioner, palliative home care allowance, healthcare service utilization in the last week of life, and public healthcare expenditures. The findings reveal that the average total healthcare expenditure for cancer patients was 68% higher in the last month and 71% higher in the last 6 months than non-cancer patients. Moreover, the authors’ results show that public expenditure was lower for the oldest persons than for younger ones, but was also closely related to the place of death (Table 1).

Other authors, like Parsonnet et al. (1996) [5], estimate the cost and effectiveness of screening for *Helicobacter pylori*, in order to prevent gastric cancer. The authors take into account the rate of incidence and prevalence (percent of gastric cancer in distal stomach, percent of patients with gastric cancer who died, life expectancy after diagnosis, prevalence of *H. plyori* infection in patients over 50 years old, and relative risk of cancer in persons with the *H. plyori* infection), screening and treatment (age, frequency, sensitivity, specificity, and treatment efficacy), cost variables (screening cost, *H. plyori* treatment cost, incremental cost), and other economic or cost-efficacy variables (annual discount rate for costs and benefits, and efficacy of prevention strategy). Parsonnet et al. (1996) [5] results show that screening and treatment for the *H. pylori* infection is potentially cost-effective in the prevention of gastric cancer, particularly in high-risk populations. Davidoff et al. (2013) [6], through a 4-year cohort construction between 1997 and 2007, demonstrates that associated cancer costs may be seen as a burden, especially for elderly patients who may not be able to afford getting treatment for their chronic illness. 

As we can see, many specialists in the field recognize the cost of cancer and its development over time as a reflection of progress in cancer care, apart from its epidemiological determinants [5,6,7]. Also, the cost of medical technology has increased over time, especially in the cancer care sector, which raises complex questions related to its effects, not only on the economy but on the citizens as well. Our overall goal is directed on framing healthcare expenditure in general, and expenditure on cancer and cancer drugs in particular, in order to underline the main differences in health spending within several European Member states.

## 2. Case Study: Healthcare Expenditures and the Cost of Cancer in European Member States

Disparities in healthcare systems in general, and in cancer care in particular (though the cancer care we refer to means the health system expenditure on care, for those with cancer as well as on the prevention and treatment of cancer), are subjects of increasing concern to the medical community. The main causes for diagnosis, treatment, and outcome disparities are very complex, and include economic, cultural, and social factors. For instance, socioeconomic factors like income and healthcare insurance coverage can influence the access to care, appropriate early disease detention, treatment, and palliative care. Several authors’ analyses show that in low-income countries, there is a lack of viable business models for cancer care, which is leading to possible misapplication or misallocation of available resources, as well as to under-performing medical care services [8]. Culture and cultural factors play an important role in modeling health behaviors, such as attitudes towards illness, or traditional beliefs of healing versus modern forms of medicine [9]. Also important, both in rich and poor countries, is the so called “healthcare waste”, which includes both general waste (sharps, non-sharps, blood, chemicals, body parts, pharmaceuticals, medical devises, and radioactive materials) and hazardous materials, which could lead to infestations, infertility, hormonally-triggered cancers, and so forth, if they are not managed properly. Lack of awareness, inadequate training, or lack of disposable waste systems and proper management may be associated with adverse health outcomes and inefficient use of financial resources [10]. On the other hand, one of the major challenges of the healthcare system is not only to preserve or improve the quality of care, but also to not deepen—and perhaps even reduce—the existing disparities in care. Moreover, healthcare expenditures are increasing at a faster rate than the global economy, and neither high- nor low-income countries will be able to manage the costs without a radical revision of price definition with companies [11]. Although many strategies aiming to control national healthcare expenses have been adopted over time, two cost strategies—*demand-side*, where patients must pay more in copayments and deductibles, and *supply-side*, which seeks to alter the incentives of health care workers to provide certain services—major discrepancies still exist across countries [12].

Other studies underline the effects of the financial and economic crisis on healthcare expenditure, through austerity measures and budget cuts. For instance, a 1% rise in unemployment was associated with an increase in mortality for several cancer subtypes: prostate cancer, breast cancer in women, lung cancer in men, and colorectal cancer in both men and women [13]. Such differences could explain at least in part the variation noted across the 28 EU member states.

This paper aims at analysing the healthcare expenditures and cost of cancer care across 28 European Union member states: Austria, Belgium, Bulgaria, Croatia, Cyprus, Czech Republic, Denmark, Estonia, Finland, France, Germany, Greece, Hungary, Ireland, Italy, Latvia, Lithuania, Luxembourg, Malta, The Netherlands, Poland, Portugal, Romania, Slovakia, Slovenia, Spain, Sweden, and the United Kingdom. 

The main indicators taken in account are: (1) health care expenditure on all types of care, which includes total expenditure (millions of euro), general government expenditure on health (percent total healthcare expenditure (THE)), total health expenditure (percent of GDP), healthcare expenditure per capita and healthcare expenditure per capita purchasing power parity (PPP); and (2) health care expenditure on cancer and anti-cancer drugs (according to ICD-10 C00D48 and ICD-10 C00-97, ICD—International Statistical Classification of Diseases and Related Health Problems), including public and private healthcare expenditures on cancer calculated as percentage of total sum of public and private expenditures, total health expenditures on cancer healthcare services, health care expenditures on cancer per capita, healthcare expenditures on cancer per capita in purchasing power parity (PPP), and healthcare expenditures on anti-cancer drugs. The cancer, in the calculation of health expenditures on cancer, is defined as neoplasms, according to ICD-10 C00D48, and according to ICD-10 C00-97, malignant neoplasms (ICD—International Statistical Classification of Diseases and Related Health Problems). The programs used for our analysis are SPSS Statistics V20.0 (IBM Corporation, Armonk, NY, USA) and Stat World Explorer.

The health gap between countries has persisted, and in some cases, has increased in recent years across the European Union. The direct costs per capita vary across the EU, with countries such as Bulgaria, Romania, Estonia, Latvia, Lithuania, etc. allocating less than 100 euro per capita on cancer drugs, compared with Austria, Belgium, France, Germany, Luxemburg, the Netherlands, and Sweden, who assign more than double that amount. However, the state of healthcare influences not only the individuals’ level of productivity, but also determines the welfare level of a country. According to the World Health Organisation (2016) [3], poor health may have devastating effects on one country’s economic development; also, the high costs of medical services may affect which patients are getting treatment. Several other studies found similar results. For instance, Bhargava, et al. [14] in 2001 investigated the effects of health (such as the adult survival rate) on economic growth for up to five-year periods, from 1965 and 1990. The results point out that although the health of individuals in a country can only be roughly approximated by national averages, the models show significant effects of adult survival rate (ASR) on economic growth for low-income countries. The effects show, for instance, that for the poorest countries a 1% change in ASR was associated with an approximate increase of 0.05% in economic growth rate [14]. The workforce’s productivity is closely linked to its healthcare status. Moreover, from an economic point of view, inadequate costs may have significant implications for overall healthcare expenditures. For instance, inadequate treatments, poorly-managed chronic conditions, or missed diagnoses can result in higher subsequent healthcare costs, which will be perceived not only by the beneficiaries, but by taxpayers who support healthcare programs as well [15].

Table 2 describes minimum and maximum values, mean, and standard deviation for public expenditure on health (as percent of total healthcare expenditure (THE)), total health expenditures calculated as percentage of GDP, healthcare expenditure per capita and healthcare expenditure per capita in purchasing power parity (PPP), and total expenditure on healthcare (millions of euro) in 28 European countries (2010 and 2014).

Country-specific data on health expenditures were gathered from reports and studies from the Knoema database and World Bank estimates [16,17].

Healthcare systems, in general, are established and financed in different ways across the European Union. In 2014, the highest level of general public (government) expenditures on healthcare was registered in the Netherlands (87% of total healthcare expenditures), followed by Denmark (84.8%), Czech Republic (84.5%), Sweden (84%), and Luxemburg (83.9%). The lowest values were registered in Greece (61.7%), Bulgaria (54.6%), and Cyprus (45.2%). In comparison to 2010, public expenditures have increased between 5% to 10% in countries like Malta, Slovakia, and Latvia, while Spain, Lithuania, Cyprus, and Croatia register the higher decrees in healthcare expenditure (%THE).

**Figure 1:**
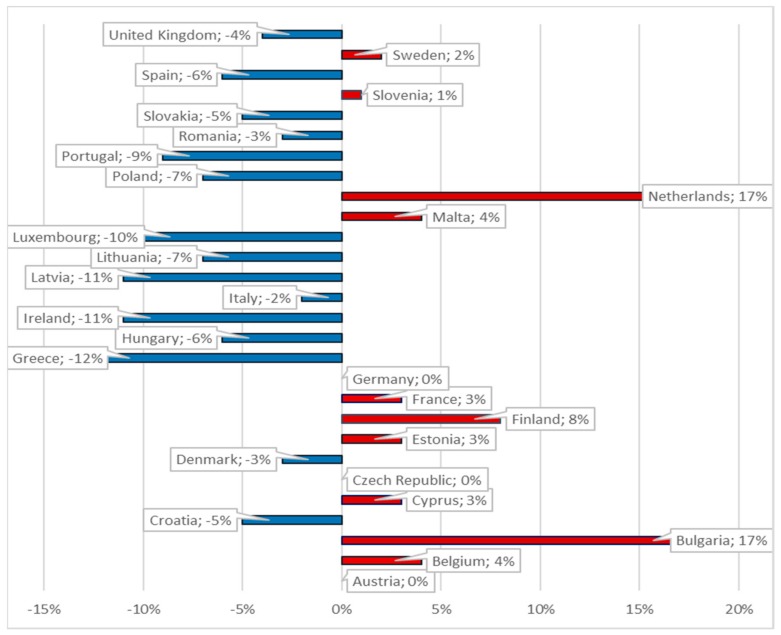
Healthcare expenditure (% of GDP) percentage change in 2010 and 2014.

In 2014, half of the European Union member states allocated 9% or more of their GDP on healthcare expenditures. Germany recorded the highest ratios of current healthcare expenditures to GDP (11.3%), followed by Austria (11.2%), Denmark (10.8%), Belgium (10.6%), and Finland (9.7%). However, healthcare expenditures accounted for less than 7% of the GDP in Luxemburg, Lithuania, Estonia, Poland, and Latvia, with Romania recording the lowest ratio (5.6%) (Figure 1). In contrast, in 2010, the highest increase was registered in Bulgaria and the Netherlands, while in Greece, Ireland, and Latvia the healthcare expenditures (percent of GDP) decreased by more than 10%. In 2014, Sweden and Malta increased healthcare expenditures per inhabitant by more than 40%. From 2010–2014, these other countries registered expenditure increases between 20–40%: Latvia, Lithuania, Bulgaria, and Estonia. On the contrary, the Czech Republic, Italy, Spain, Croatia, Cyprus, Portugal, and Greece diminished their per capita expenses in 2014, from 2% up to 29%, in comparison with 2010. The overall mean increased in the EU 28, from 2183.39 to 2348.18 euro per capita in four years. In 2014, Luxemburg, Sweden, Denmark, Netherlands, Austria, and Germany allocated more than 4000 euro per inhabitant. A mid-range ratio between 2000 Euro and almost 3750 Euro per inhabitant was recorded in France, Belgium, Finland, Ireland, the United Kingdom, Italy, and Spain. At the other end of the spending scale (less than 1000 Euro per capita) are Estonia, Lithuania, Croatia, Hungary, Latvia, Poland, and Bulgaria, with Romania recording the lowest value (419.92 euro).

Health care expenditure per capita in purchasing power parity expresses the disparities among European countries, after adjusting for price differences, by calculating expenditure in purchasing power parity. Luxemburg occupies the first position, followed by Sweden, the Netherlands, Germany, Austria, Denmark, France, and Belgium, each with PPP values greater than 3.3 thousand euro per inhabitant. Healthcare expenditures per capita in purchasing power parity values between 2 and 3 thousand euro per inhabitant are in Ireland, Finland, United Kingdom, Italy, Malta, Spain, Slovenia and Portugal. Romania (813.46 Euro) and Latvia (708.67 Euro) have the lowest values from the 28 EU countries. The overall mean increased after price adjustment, from 2152.42 in 2010 to 2342.95 in 2014. 

Total healthcare expenditures sums up public and private healthcare expenditures, healthcare services, activities related to family planning, nutrition, and emergency aid. The highest total healthcare expenditures are registered in Germany, followed by France, the United Kingdom, Italy, and Spain. Between 20.00–40.00 million euro in 2014 were allocated in Austria, Denmark, and Poland, each, while the lowest values were registered in Cyprus (1176 million euro) and Malta (791.59 million euro). As the analysis shows, some of European Union member countries continue to battle with high and rising costs of healthcare. Although expenditures on new medical devices, products, and procedures can improve the quality and prolong life, the Institute of Medicine (2003) [18] stated three main reasons why a cost reduction may also be taken into account: (1) additional healthcare spending has significant opportunity costs; (2) market imperfection may lead to more spending in the healthcare system—first, through the healthcare services provided (for instance healthcare insurance), many consumers never face the medical goods and services’ true costs, and second, due to limited information consumers tend to buy medical goods based on advertising, not on their true needs; (c) government spending for healthcare (percent of GDP) has increased, although the size of active populations have decreased in many European countries.

Table 3 describes healthcare expenditures for cancer through the following indicators: public and private healthcare expenditures on cancer, calculated as percentage of total sum of public and private expenditures; total health expenditures on cancer healthcare services; healthcare expenditures on cancer per capita. We based our analysis on Jönsson et al. [7] estimates on the development of different types of healthcare expenditures on cancer care. The expenditures on cancer are calculated in a top-down manner, having as a starting point the country’s GDP, which was multiplied by the share of total GDP expenditures on healthcare. Healthcare expenditures on healthcare (total) is multiplied by an estimate of the share of cancer-specific health expenditures, in order to determine the healthcare expenditure on cancer. The data was gathered from different databases, reports, etc. for 17 countries, while for the remaining 11 estimates were realized based on geographical proximity and GDP per capita similarity [6]. 

In the analysis, cancer is defined as neoplasms (according to ICD-10 C00-D48) in the calculation of health expenditures on cancer, and malignant neoplasms (according to ICD-10 C00-9) in the calculation of production loss due to premature mortality and epidemiological development (see Appendix A).

**Figure 2:**
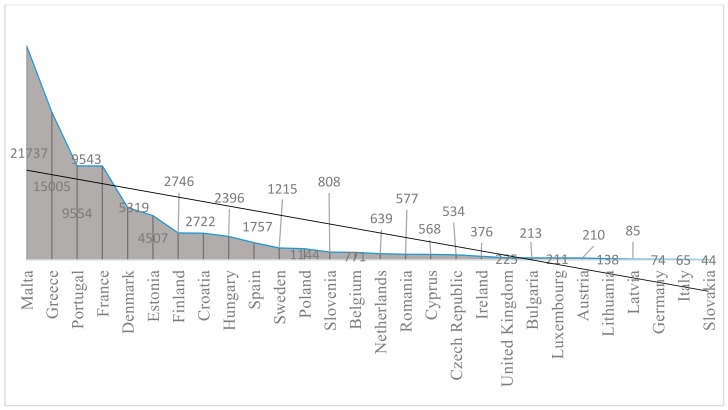
Total health expenditures on cancer: current prices for the 28 EU member states, 2014.

Cancer represents a major healthcare challenge, not only within European Union member countries, but worldwide. Cancer, despite constant improvement in care, mortality, and survival, has a tremendous human cost in every country, and it may place an increasing burden on countries—including not only medical care costs, but also expenses on non-medical care, placing additional weight on patients and their caregivers [19]. According to the data provided by Jönsson [7], healthcare expenditures on cancer in the European Union member states represented roughly 6% of total healthcare expenditures in 2014. The values ranged from 3.9% in Portugal to 7% in Hungary (Table 3). High values were registered also in Croatia (6.9%), Austria (6.8%), Bulgaria (6.8%), Germany, and Romania (6.8%). Values lower than 5% were also registered in Denmark and Finland. Total health expenditures on cancer reached their highest values in Malta, Greece, Portugal, and France, while the lowest were registered in Germany, Italy, and Slovakia (Figure 2). 

**Figure 3:**
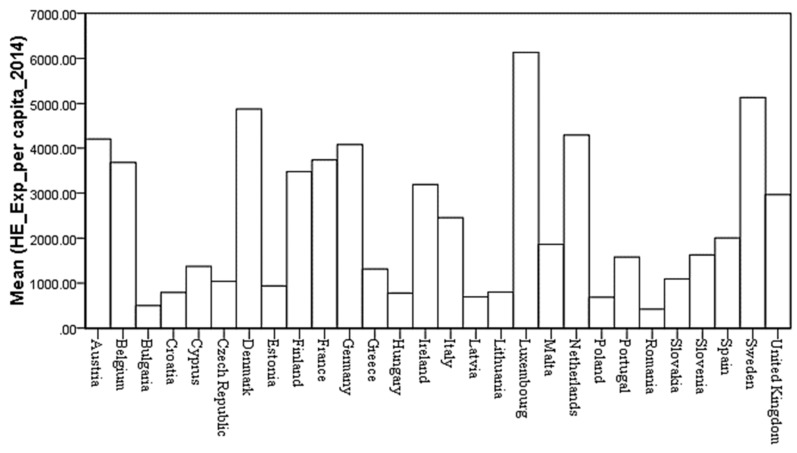
Health expenditures on cancer (per capita) for the 28 EU member states, 2014.

High disparities in healthcare spending on cancer per inhabitant were observed across European states. The highest expenditure per capita is registered in Luxemburg (379 euro), while Denmark, Ireland, Italy, Finland, and the United Kingdom allocate less than half that amount. High values (above 200 euro) were also registered in Sweden, Austria, the Netherlands, Germany, Belgium, and France. The lowest values were registered in low-income countries like Bulgaria (29 euro) and Romania (27 euro) (Figure 3). After price differential adjustments, Central and Western Europeans registered healthcare expenditures of over 200 euro on cancer per inhabitant, while in Southern Europe (except Portugal and Croatia), per capita expenses were between 100 and 155 euro. Latvia (64 euro) and Romania (53 euro) registered the lowest values.

Another important indicator in analyzing European Union healthcare expenditures on cancer is the amount spent on anti-cancer drugs. In the literature, several studies have shown the deep financial impact related to the spread of problems posed by a cancer diagnosis [20]. Even in the insured patients’ cases, costs associated with cancer care may still be substantial, and can represent a barrier in receiving high-quality care (personal bankruptcy, savings loss, or treatment delays). The increasing cost of cancer treatments and diagnoses impact stakeholders and patients alike. The main reasons identified in the literature for high costs associated with cancer care are, namely, (a) the need for clinical trials and approvals, compound identification or cancer drug designing, the risks associated with the development, production, and marketing of new drugs and technologies (advances in imaging, or robotics applied to surgery or therapeutic radiology), as well as highly specialized human resources may reflect a larger amount of resource spending [2,21,22]; (b) most cancer types are incurable, and anti-cancer drug beneficiaries are treated with each approved agent, creating a virtual monopoly, because patients receiving chemotherapy treatments will still die of their disease. In this scenario, there is no competition among truly effective cancer drugs to lower their costs [21]; (c) once a new and improved anti-cancer drug is released, the older version is seen as substandard treatment [17]; (d) even for very small improvements in the outcome, patients, alongside physicians, are often prepared to pay the drugs’ high cost, or they assign a higher value to cancer drugs [2,21].

Vogler and Vitry [23], through a cross-country price comparison study, reveal great disparities between cancer drugs across 18 countries (16 from the European Union, Austria, and New Zeeland). High differences (from 28–388%) were found, especially in countries like Greece, where prices ranked at a low level, whereas in Sweden, Switzerland, and Germany, price data showed similarly high ranges. 

Other authors, like Wilking and Jönsson [24], consider that the cost of cancer drugs can be estimated in absolute terms, in relation to the healthcare spending or in relation to total drug spending. Wilking and Jönsson’s estimation included 19 European countries, and the cost for cancer drugs were calculated for the 2002–2003 period. Their results reveal that for the 19 EU countries, the total expenditures on cancer drugs were approximately 4.5 billion euro in 2002 (ex-factory prices). The highest values were registered in France and Germany, which spent more than 950 million euro on cancer drugs in 2002. Ireland, on the other hand, spent the lowest amount on cancer drugs (28 million euro); thus, cancer drugs per capita accounted for only 7 euro in that country [24].

**Figure 4:**
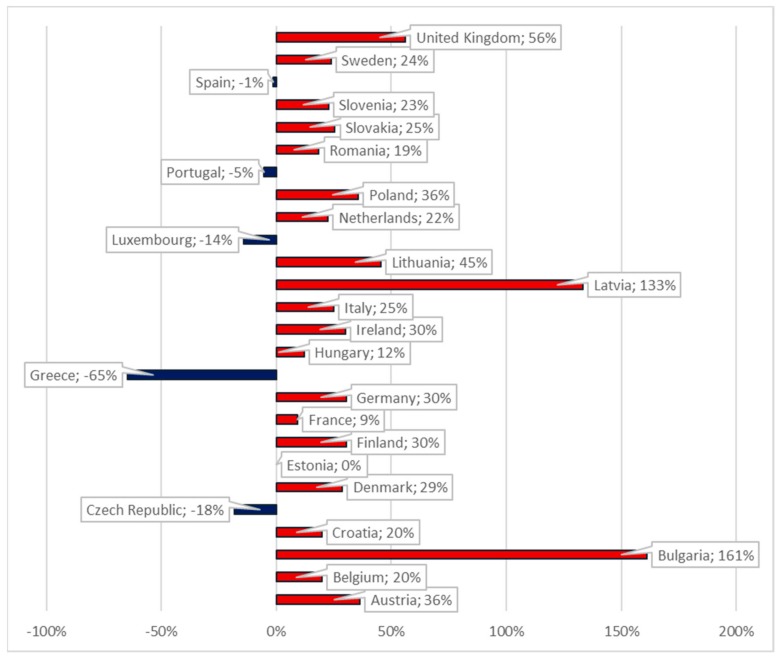
Expenditures of anti-cancer drug percentage change, 2010 and 2014 (26 EU countries).

Figure 4 illustrates the percentage change in anti-cancer drugs expenditures across four years (2010–2014) in 26 European countries. We constructed our analysis on Jönsson et al.’s [7] estimates of the development of cancer drug costs. The expenditure on anti-cancer drugs was calculated based on country-specific data for drug sales to hospitals and retailers, obtained from Jönsson et al.’s [7].

The highest percentage change was registered in Latvia and Bulgaria, with an increase of more than 130% in four years. Of the 26 European countries studied, above-average changes were also registered in Denmark (29%), Finland, Germany, and Ireland, all three with an increase over 30%, as well as Austria (36%), Poland (35%), Lithuania (45%), and the United Kingdom (56%). From the analyzed countries, five have registered decrees in anti-cancer drug expenditure: Spain, Portugal, Luxemburg, Czech Republic, and Greece.

In absolute terms, the average mean expenditures in the 26 EU countries have increased from 600 million to more than 733 million euro between 2010 and 2014. 

**Figure 5:**
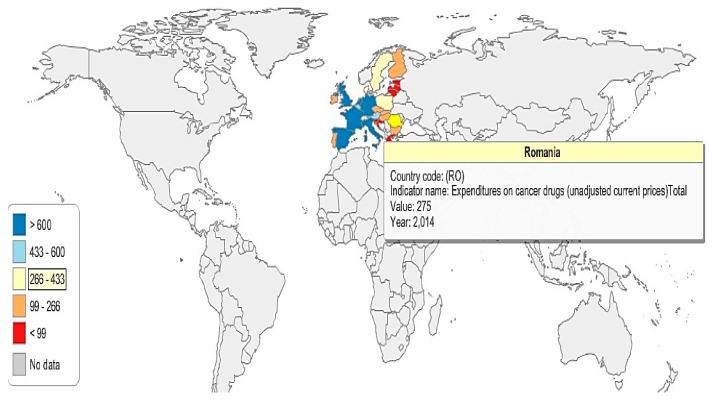
Anti-cancer drug expenditure map, Romania and the 26 EU countries, 2014.

**Figure 6:**
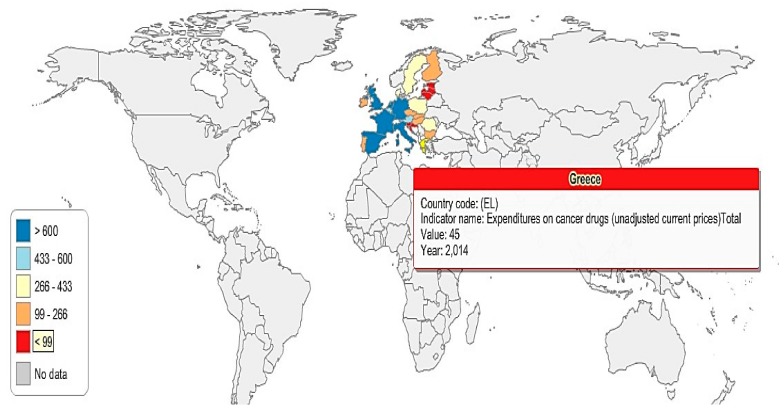
Anti-cancer drug expenditure map, Greece and the 26 EU countries, 2014.

In order to have a better image of the 26 EU countries’ (due to lack of data, Cyprus and Malta were excluded from the analysis) expenditures on anti-cancer drugs, five groups have been created (G1, G2, G3, G4, and G5), according to the amount of financial resources spent on cancer drugs (absolute values) (Figure 5 and Figure 6):

- G1: The Netherlands, Spain, the United Kingdom, Italy, France, and Germany;
- G2: Austria and Belgium;
- G3: Poland, Sweden, Romania, and Denmark;
- G4: Hungary, Portugal, Finland, Ireland, Czech Republic, Slovakia, and Bulgaria;
- G5: Croatia, Slovenia, Greece, Lithuania, Latvia, Estonia, and Luxemburg.

In 2014, the highest expenditures on cancer drugs were registered in the Netherlands, Spain, the United Kingdom, Italy, France, and Germany (G1), where anti-cancer expenditures exceed €600 million. Austria and Belgium (G2), spent between 433–600 million euro in 2014 on anti-cancer drugs; meanwhile, Poland, Sweden, Romania, and Denmark’s expenditures were situated between 266–433 million euro in the same year (Figure 5 and Figure 6). 

G4 represents the countries that allocated between 99–266 million euro on cancer drugs in 2014, while the countries in G5 are those who spent less than 99 million euro on anti-cancer drugs. 

## 3. Discussion and Conclusions

The overall picture confirms that there were considerable disparities between the 28 countries with relation to their expenditure on health. There is a wide range of variation of healthcare expenditure across the 28 EU member states. The trend in public expenditures for all types of care, compared to the share of health expenditures as a GDP percentage, shows the increase of health expenses across 2010 and 2014; however, it is a lower increase compared to the overall GDP increase. With regards to healthcare expenditures related to population size, poorer countries devoted smaller amounts (less than 1 thousand euro) than the wealthier countries (above 3.5 thousand euro). Even after adjusting for price differences, although the overall disparity level was lower, the ratio between the highest and the lowest levels of healthcare expenditures per capita was 5 to 1. Other countries though, like the United States, that spend a large proportion of their GDP on healthcare also experience high rates of disease, injury, health-damaging behaviors, and devastating results on cancer survivors. The main arguments behind these results include differences in healthcare, individual behaviours (such as tobacco use, obesity, diet, etc.), socioeconomic inequalities, and the physical and built environment (urban design, transport infrastructure, food environment, etc.). On the other hand, there are countries that spend a small proportion of GDP on healthcare, but also do not have good health outcomes (some developing countries, especially sub-Saharan Africa (SSA)). The majority of countries across developing regions depend mostly on loans to finance healthcare, which has proved to be not only inadequate, but also unsustainable for covering the healthcare burden.

Cancer represents a major healthcare challenge, not only within European Union member countries, but worldwide. Cancer, despite constant improvement in care, mortality, and survival, has a tremendous human cost in every country, and it may place an increasing burden on countries, which includes not only medical care costs, but also expenses on non-medical care, placing additional weight on patients and their caregivers. In the 28 EU member countries, healthcare expenditure on cancer (%THE) is rather low, despite the high cost associated with anti-cancer drugs. New treatments and drug development is increasingly difficult to achieve if the share devoted to cancer does not increase, and it may act as a barrier in receiving high-quality care. Also, the lack of targets or expectations for value makes the development of new cancer drugs dependent mostly on the price of recently-marketed cancer drugs. In highly dysfunctional markets, the perverse incentives make it impossible for the drug prices to reflect their given value. Higher costs of drugs are mostly justified through the associated risky development, which is necessary for achieving success. However, the costs associated with product development are passed on to the consuming public, as the government measures assure some kind of safety net for those manifesting a high-risk endeavor [25]. An efficient behavior targeted towards removing perverse incentives and adopting improved rules of the game, and capable of assuring a proper functioning of the market systems, is necessary for achieving high-quality care. 

In this paper, we have used two sets of measurement variables: (1) healthcare expenditures on all types of care, which include total expenditures (millions of euro), general government expenditures on health (percentage of total healthcare expenditure (THE)), total health expenditure (percent of GDP), healthcare expenditure per capita and healthcare expenditure per capita purchasing power parity (PPP); and (2) health care expenditure on cancer and anti-cancer drugs, including both public and private healthcare expenditures on cancer, calculated as a percentage of the total sum of public and private expenditures, as well as total health expenditures on cancer healthcare services, healthcare expenditures on cancer per capita, healthcare expenditures on cancer per capita in purchasing power parity (PPP), and healthcare expenditures on anti-cancer drugs. However, this approach could be expanded through integrated additional variables and a larger sample of countries. For instance, a comparison of cost-effective spending in developing and developed countries could provide useful insights on the quality of healthcare, especially on cancer care.

Another direction for further research may include the effects of the economic crises, through austerity measures, higher unemployment, decreased public expenditures, etc. on cancer mortality and the overall quality of the healthcare system.

## Figures and Tables

**Table 1:** Summary of studies concerning cancer costs.

Author (s)	Method/Sample	Location	Statistical Procedure	Outcome (s)	Summary of Findings
Bernand et al., 2011 [1]	Panel Survey (18–64 years persons)	US	Five rounds of interviews	high risk burden is more associated with cancer patients compared with other chronically illnesses	high burdens/costs may affect treatment choice and deter patients from getting care
Gielen et al., 2010 [4]	Quantitative analysis performed in SAS (version 9.1) on 40,974 individuals (age ≥ 40 ears)	Belgium	Regression analysis (multinominal and linear) and analysis of variance	end of life for older persons, especially aged ≥ 90 years differs from that of younger generations	public expenditure was lower for oldest persons as for younger generation but closely related to the place of death
Parsonnet et al., 1996 [5]	Mixed approach (Markel model implementation and base-case analysis 5-years screening of patients from 50–54 years)	US	Qualitative review; Sensitivity and costeffectiveness analysis	cost-effectiveness was sensitive to the efficacy of the cancer prevention strategy	Screening and treatment for *H. pylori* infection is potentially cost-effective in the prevention of gastric cancer, particularly in high-risk populations.
Davidoff et al., 2013 [6]	Retrospective, observational study (~4500 new beneficiaries/year (1997–2007) who remain in the survey for up to 4 years)	-	Linear models and logistic regressions; 4-year cohort construction	protection against out of pocket burden can be achieved through supplemental insurance and higher income	Higher level of out-of-pocket burden is associated with cancer medical benefices; financial burden may affect elderly patients for getting treatment

**Table 2:** Health care expenditure on all types of care (28 European Union (EU) member states), 2010 and 2014.

	Year	2010				2014			
Indicators		Minimum	Maximum	Mean	Std. Deviation	Minimum	Maximum	Mean	Std. Deviation
Pub_exp (%THE): General public expenditures on healthcare, calculated as percentage of total sum of public and private expenditure.		47.40	86.70	73.77	9.57	45.20	87.00	73.39	9.86
HE_exp (%GDP): Total health expenditures (public and private expenditures on healthcare), calculated as a percentage of GDP.		5.80	11.70	8.78	1.66	5.60	11.90	8.66	1.79
HE_Exp_per capita: Total health expenditures, calculated as a ratio of total population. Data are in current Euro terms.		367.37	6019.99	2183.39	1494.73	419.92	6135.24	2348.18	1649.28
HE_Exp_per capita_PPP: Total health expenditures, calculated as ratio of total population. Data are in current Euro terms.		608.50	4928.47	2152.42	1048.88	708.67	5135.57	2342.95	1161.25
HE_Exp_Total: Total health expenditures, as the sum of public and private health expenditures on healthcare services, activities related to family planning, nutrition, and emergency aid (euro).		548.78	290,625.41	45,524.26	74,118.65	791.60	329,455.05	49,205.19	82,107.98

**Table 3:** Health care expenditure on cancer (28 EU member states), 2014.

Indicator	Description	Minimum	Maximum	Mean	Std. Deviation
HE_Exp_on cancer (% of THE)	Public and private healthcare expenditure on cancer calculated as percentage of total sum of public and private expenditure.	3.90	7.00	6.06	0.83232
HE_Exp_on cancer (Total)	Total health expenditure on cancer	44.00	21,737.00	2970.82	5131.60428
HE_Exp_on cancer (per capita)	Health expenditure on cancer calculated as ratio of total population. Data are in current Euro.	27.00	379.00	136.85	97.10083
HE_Exp_on cancer (per capita_PPP)	Health expenditure on cancer calculated as ratio of total population Data are in euros converted using 2005 purchasing power parity (PPP) rates.	53.00	311.00	138.82	69.54777

## Appendix A

Cancer is defined, according to ICD-10 C00-D48, as neoplasms, in the calculation of healthcare expenditures on cancer:

D48.0 Neoplasm of uncertain behavior of bone and articular cartilage;
D48.1 Neoplasm of uncertain behavior of connective and other soft tissue;
D48.2 Neoplasm of uncertain behavior of the peripheral nerves and autonomic nervous system;
D48.3 Neoplasm of uncertain behavior of the retroperitoneum;
D48.4 Neoplasm of uncertain behavior of the peritoneum;
D48.5 Neoplasm of uncertain behavior of the skin;
D48.6 Neoplasm of uncertain behavior of the breast;
  - D48.60 Neoplasm of uncertain behavior of an unspecified breast
  - D48.61 Neoplasm of uncertain behavior of the right breast
  - D48.62 Neoplasm of uncertain behavior of the left breast
D48.7 Neoplasm of uncertain behavior of other specified sites;
D48.9 Neoplasm of uncertain behavior, unspecified.

In addition, according to ICD-10 C00-97, cancer is defined as malignant neoplasms, in the calculation of production loss due to premature mortality and the epidemiological development.

## References

[1] Bernard D.S., Farr S.L., Fang Z. (2011). National estimates of out-of-pocket health care expenditure burdens among nonelderly adults with cancer: 2001 to 2008. J. Clin. Oncol..

[2] Meropol N.J., Schrag D., Smith T.J., Mulvey T.M., Langdon R.M., Blum D., Schnipper L.E. (2009). American Society of Clinical Oncology guidance statement: The cost of cancer care. J. Clin. Oncol..

[3] World Health Organization Metrics: Disability-Adjusted Life Year (DALY). http://www.who.int/healthinfo/global_burden_disease/metrics_daly/en/.2016.

[4] Gielen B., Remacle A., Mertens R. (2010). Patterns of health care use and expenditure during the last 6 months of life in Belgium: Differences between age categories in cancer and non-cancer patients. Health Policy.

[5] Parsonnet J., Harris R.A., Hack H.M., Owens D.K. (1996). Modelling cost-effectiveness of Helicobacter pylori screening to prevent gastric cancer: A mandate for clinical trials. Lancet.

[6] Davidoff A.J., Erten M., Shaffer T., Shoemaker J.S., Zuckerman I.H., Pandya N., Stuart B. (2013). Out-of-pocket health care expenditure burden for Medicare beneficiaries with cancer. Cancer.

[7] Jönsson B., Hofmarcher T., Lindgren P., Wilking N. (2016). The cost and burden of cancer in the European Union 1995–2014. Eur. J. Cancer.

[8] De Souza J.A., Hunt B., Asirwa F.C., Adebamowo C., Lopes G. (2016). Global health equity: cancer care outcome disparities in high-, middle-, and low-income countries. J. Clin. Oncol..

[9] Ward E., Halpern M., Schrag N., Cokkinides V., DeSantis C., Bandi P., Jemal A. (2008). Association of insurance with cancer care utilization and outcomes. CA A Cancer J. Clin..

[10] Oli A.N., Ekejindu C.C., Adje D.U., Ezeobi I., Ejiofor O.S., Ibeh C.C., Ubajaka C.F. (2016). Healthcare waste management in selected government and private hospitals in Southeast Nigeria. Asian Pacif. J. Trop. Biomed..

[11] Mustacchi G., Generali D. (2017). Cost-effectiveness and sustainability of breast cancer screening and new anti-cancer drugs. J. Med. Econ..

[12] Ellis R.P., McGuire T.G. (1993). Supply-side and demand-side cost sharing in health care. J. Econ. Perspect..

[13] Maruthappu M., Watkins J., Noor A.M., Williams C., Ali R., Sullivan R., Atun R. (2016). Economic downturns, universal health coverage, and cancer mortality in high-income and middle-income countries, 1990–2010: A longitudinal analysis. Lancet.

[14] Bhargava A., Jamison D.T., Lau L.J., Murray C.J. (2001). Modeling the effects of health on economic growth. J. Health Econ..

[15] Vodă A.I., Ţigănaș C.G. (2015). Healthcare Quality and Its Effects on Growth. A Regional Analysis. CES Work. Papers.

[16] Knoema (2017). Knoema Database. https://knoema.com/atlas.

[17] World Bank Database (2017). Washington. http://data.worldbank.org/.

[18] Institute of Medicine (2003). Unequal Treatment: Confronting Racial and Ethnic Disparities in Healthcare.

[19] OECD (2013). Cancer Care: Assuring Quality to Improve Survival.

[20] Ward E., Jemal A., Cokkinides V., Singh G.K., Cardinez C., Ghafoor A., Thun M. (2004). Cancer disparities by race/ethnicity and socioeconomic status. CA A Cancer J. Clin..

[21] Siddiqui M., Rajkumar S.V. (2012). The high cost of cancer drugs and what we can do about it. Mayo Clin. Proc..

[22] Dickson M., Gagnon J.P. (2009). The cost of new drug discovery and development. Discovery Med..

[23] Vogler S., Vitry A. (2016). Cancer drugs in 16 European countries, Australia, and New Zealand: A cross-country price comparison study. Lancet Oncol..

[24] Wilking N., Jönsson B. (2005). A pan-European comparison regarding patient access to cancer drugs. Karolinska Instit. Stockh. Sch. Econ..

[25] Saltz L.B. (2016). The Value of Considering Cost, and the Cost of Not Considering Value. J. Clin. Oncol..

